# The impact of liquidity risk and credit risk on bank profitability during COVID-19

**DOI:** 10.1371/journal.pone.0308356

**Published:** 2024-09-09

**Authors:** Muhammad Haris, HongXing Yao, Hijab Fatima

**Affiliations:** 1 School of Finance and Economics, Jiangsu University, Zhenjiang, China; 2 Institute of Banking & Finance, Bahauddin Zakariya University, Multan, Pakistan; American University of the Middle East, KUWAIT

## Abstract

The COVID-19 outbreak caused a massive setback to the stability of financial system due to emergence of several other risks with COVID, which significantly influenced the continuity of profitable banking operations. Therefore, this study aims to see that how differently the liquidity risk and credit risk influenced the banking profitability during Covid-19 (Q12020 to Q42021) than before COVID (Q12018 to Q42019). The study employs pooled OLS, and OLS fixed & random effects models, to analyze the panel data on a sample of 37 banks currently operating in Pakistan. The results depict that liquidity risk has a positive and significant relationship with return on assets and return on equity, but insignificant relationship with net interest margin. Credit risk has a negative and significant relationship with return on assets, return on equity, and net interest margin. The study also applies quantile regression to address the normality issue in data. The quantile regression results are consistent with pooled OLS, and OLS fixed and random effects results. The study makes valuable suggestions for regulators, policymakers, and others users of financial institutional data. The current study will help to set policies for efficient management of LR and CR.

## 1. Introduction

A stable financial system is indispensable for an inclusive economy and within the financial system the banking sector plays a key role [1]. Since its inception from 1947, the Pakistani financial-sector has experienced numerous challenges, including lack of capital and political instability, which have hampered the country’s industrial growth [2]. Pakistan is one of few countries who have been ruled by the blend of military and democratic governments. So far, 77 years old Pakistan has been ruled by thirty-seven governments (military, interim and elected) and almost 167 political parties have been registered since its independence (Aug-1947). No elected prime minister in Pakistan has ever completed his full term. As a results, the legacy of stable economic policies never continued in Pakistan. Pakistani banking sector has been experienced a nationwide nationalization in 1974 by a democratic government, while based on the consequent results the financial sectors again experienced the denationalization in 1980 by a military government. Many studies have been reported that the profitability and efficiency of Pakistani banking sector has been hit hard due to the rapid changes in economic policies because of swift change in country’s governance [3–5]. It is an undoubtable fact that the efficiency and profitability of banking sector is essential for the economic development. However, emergence of different crises possess serious threats to the stability of financial system. Since the outbreak of COVID-19, the banking sector around the globe experienced abrupt supply of deposits and recovery loan repayments, which created the concerns about liquidity and credit risks. Likewise, the profitability of the banking industry has also been affected. This effect of COVID on the banks profitability was not retained to a single country only but it was scattered to the global financial sector since early 2020s, and thus lead countries to declining in their economic activities. The period of COVID-19 pandemic has weakened the stakeholders trust in their ability to ensure effective management of risk in financial institutions. The pandemic had disrupted the global trade and financing, disordered bulk markets, reduced consumptions, savings, and exports [6]. The COVID-19 crisis had also disturbed all aspects of the performance of banking sector. Similarly, Ghenimi, Chaibi [7] reported that the COVID-19 significantly affected the profitability of banks in MENA region. Therefore, this study aimed to examining how liquidity and credit risk affected the profitability of banking sector in Pakistan. The focus on Pakistan in this study is because that, among only few countries around globe, Pakistan is the one who managed the COVID-19 very successfully by imposing the smart lockdown, introducing loan restructuring schemes and offering the subsidized interest rates in order to keep the smooth economic wheel.

The COVID-19 has turned the global trade activities under lockdown. Many countries including Pakistan somehow managed the COVID-19 situation better by imposing the smart lockdown for months. Though smart lockdown was an effective strategy for pandemic management but the cash inflows of banks dropped significantly due to closure of economic activities. On the one hand, the bank deposit ratio was dropped, which created the liquidity shortage and thus, squeezed the avenues of more funds available to be placed into earning assets such as lending’s and investments. One the other hand, loan recovery rate was dropped because of the closure of economic activities that made bank borrowers unable to repay their debts. As a result, banks had to face the loan losses situations that posed a threat not only to credit risk but also to liquidity risk. Furthermore, the value of bonds and other financial instruments declined, adversely impacting banks and further reducing their revenue [8]. The firms required more funds to meet their unavoidable expenditures because of the closure of their operational activities. As a results, the demand for loans increased was also increased. In addition, another concerning issue for the banks was shortage in the supply of many financial services, which reduced the banks’ non-interest income. Further, low returns-on-deposits, non-payment of loans by borrowers and large number of withdrawals contributed to reducing the banks’ profitability [9]. During COVID-19, a reduction in credit demands, intermediate business and an increase in non-performing loans (NPL) impacted the liquidity of banks. COVID-19 caused a desperation in manufacturers and consumption, making it difficult to collect NPLs, reduce deposits and ban industries [10]. The shrinkage investment portfolio, hitches in capital turnover and negative impact of CR affected the banks liquidity system and loan portfolio [11].

Financial institutions control and manage the liabilities, assets and risk structure, which are increasingly becoming intricate day-by-day. These are considered the important factors that affect the health of banks. Banks as the key players contribute a crucial role in economic growth and development. However, banks also face risks when providing their services. Among all, the most crucial are credit and liquidity risks. Galvis-Ciro, de Moraes [12] expressed that the political environment, rising unemployment, a high ratio of withdrawals and compression of monetary policy effect the position of liquidity risk (LR). In a sudden and unforeseen situation, when investors withdraw large amount of funds while banks have insufficient funds to fulfill those withdrawals then the risk of liquidity increases [13]. LR is one of the major causes of banks failures [14]. This risk arises when there is an excessive withdrawals without adequate reserves in hand. Diamond and Rajan [15] emphasized that LR is considered as the significant risk that causes serious threat to the banking sector. A bank fails to preserve good asset-standard, high earning and adequate capital if it has poor liquidity management. LR damages the banks repute, performance and profitability structure.

credit risks (CR) along with LR is the most common type of risks that directly affects the profitability of banks [16]. The significant relationship between CR and profitability is disclosed in literature because bank income is directly dependent on the quality of loans. The positive affiliation among profitability and CR is maintained by the expected-bankruptcy-cost hypothesis. In order to ensure the sustained future, the prospective bank administrators implement procedures that increase the bank equity to decreasing CR. CR predicted to be indispensable for the profitability position of banks [17]. Numerous existing theorists expressed the significant impact of CR on different countries [18–22]. CR discloses mostly the negative effect on banks profitability because loans are the main source of interest income for banks. The crises illustrate obstacles for banks to issue new loans and impedes profit earning. This study also intend to find the evidence and reasons for the low profitability that is faced by banks during COVID-19. The pandemic has demonstrated the importance of maintaining excess liquidity and capital as a buffer against unexpected shocks. Financial institutions with higher level of capital and liquidity buffers are better able to combat the crises and maintain profitability. According to Baret, Celner [23] banks that had higher level of capital and liquidity buffer prior to the pandemic were less likely to experience decline in profitability and were better able to support their customers during the crises.

According to the classic theory, CR and LR are related to each other but in several literature, they have been elaborating separately. However, Aydemir and Guloglu [24] found that LR and CR are not only related to each other but they are also related to the sustained profitability of the banks. Their study further elaborated the reasons of bank failures and suggested important actions that banks should perform to generate profit. The assets and liabilities of banks reported in their balance sheets are the only sources to showing their liquidity position. For the mitigation of LR, banks should manage the asset, liabilities and off-balance sheet components and carefully administer the LR management. The higher the LR, the more it can weakens the bank’s profitability, and may spillover severe effects on the survival of banks. Some previous studies found the significant impact of LR on profitability [25–28]. However, studies on the impact of LR and CR on banks profitability during COVID-19 are not yet available with clear substantiation. Therefore, our study is the first, which intended to seek an answer to the following question that what was the impact of LR on the profitability of Pakistani banks during COVID-19?

The contribution of this study is as follows. First, this study provides a comprehensive analysis on the impact of LR and CR on banks profitability using three reliable measurements of profitability, i.e., ROA, ROE and NIM [29]. Second, our study provides a comparative analysis of the impact of LR and CR on banks profitability before COVID-19 with COVID-19 period. Third, our study provides the more reliable results by using three econometric methodologies, i.e., Pooled OLS, OLS fixed and random effects, and Quantile regression. Forth, the analyses of this study are based on the quarterly data, which offered robust inference because of the lesser time-varying effects. The results of our study indicates the relationship of LR and CR with profitability of Pakistani banks during and before COVID-19. The results report that before COVID-19, the LR translates a positive but weak impact on ROA and NIM and an insignificant impact on ROE. While during COVID-19, the LR translates a strong positive impact on ROA, ROE and NIM. As for as the CR is concerned, it reports its negative impact on ROA, ROE and NIM both during and before COVID-19 periods. However, the impact of CR is not high during COVID-19 because of introducing the loan restructuring schemes with subsidized interest rates, which helped banking industry to manage the loan defaults.

The remainder of this paper proceeds as follows. Section 2 summarizes the literature review based on existing theories and studies. Section 3 presents the data and hypothesss discussion. Section 4 elaborates the empirical findings and section 5 is based on the discussion and explanation of empirical findings. Section 6 expresses the conclusion, policy implications along with recommendations future directions.

## 2. Literature review

### 2.1 Theoretical background

There are several types of risk such as liquidity, credit and markets risks, which can be triggered during any type of crises. These risks translate their extensive impact on the bank’s financial performance. Among all, the credit risk (CR) and liquidity risk (LR) are the most important because both risks are associated with the core activities of the banking sector such as lending and borrowings. The credit risk undoubtedly is the most important because banks margins are associated with loans [30]. If the quality of these loans are poor it created two effects; one, banks will suffer from lower margins and second, banks will suffer from liquidity problems because of non-repayments of loans. Thus, the economic downturn because of different crises unable the bank borrowers to make debt repayments. Therefore, the CR is found to be negatively linked with profitability during crisis period [31]. Similarly, the liquidity risk is also important. Though, the liquidity creation is the fundamental activity but the risk associated with liquidity may possess serious threat during the crisis [15, 32]. However, the nature of crisis may determine the effect of pre-crisis LR and CR on the bank profitability during a crisis [32]. During the recent financial crisis, the importance of liquidity and credit risks was highlighted more [33]. Both risks were suggested to be reduced by diversification strategy even during the crises periods [34]. The portfolio theory suggests that the risk level depends on the size of the banks. A larger bank collects funds from diversified depositors and place them to diversified borrowers, which may reduce the risk of lower liquidity and high loan defaults and thus enhances the profitability [4, 35, 36]. Similarly, it is also stated by the theory of bank lending that the larger banks in terms of deposits are less vulnerable to risk because banks with high funding liquidity risk does not make risky investments and thus keep themselves away from liquidity and credit risks [37–40]. Previous studies reported that both credit and liquidity risks jointly affect the instability of banks as both are positively linked with each other [41, 42]. Consequently, the financial intermediation theory of Bryant [43] and Diamond and Dybvig [44], which is extended by Qi [45] and Diamond [46], and the industrial organization approach to banking state that the change in the credit risk changes liquidity risk and vice versa. Hence, both risks influence the banking performance [41, 42, 47]. The liquidity risk arises as a result of reduced cash inflows because of the non-repayments of loan amount and thus, LR influences profitability [48]. A high credit risk, because of maintaining the high loan loss provisions, influence the ability of banks to meet sudden withdrawal demands. It also increases the concerns of LR and thus, influences profitability. However, the management hypothesis suggests that banks can improve their performance by joint management of credit and liquidity risks [49–53]. However, if managed well the effects of these risks might be indifferent during the time of economic downturn. For example, the credit requirements become easy during economic expansion, while these are tightened during crises in order to keep banks safe from risks. Similarly, adverse selection theory suggests that banks will not reduce the interest rates during crises because it will increase the demand for loans [54]. This increased demand for loans during crises will increase the chances of low quality loans and thus, enhances the concerns for LR and CR.

### 2.2 Empirical findings

#### 2.2.1 Findings on a single economy

Previous research has presented significant negative effects of financial risks on profitability [55–57]. Tran, Bui [58] applied fixed effects estimator on the data of 28044 U.S banks and showed that the liquidity hoarding significantly impacts banks stability. The results suggest that the correlation among plummeting earnings fluctuations, NPLs and likelihood of bankruptcy indicate improvements in stability. Viverita, Bustaman [59] explored the significant impact of liquidity in banking sector by using the sample of 85 Indonesian banks. The study suggested that the addition of assets may not be an effective approach to provoke liquidity because financial sector shifted their assets to prudent investment. However, in contrast to conventional the Islamic institutions managed liquidity very significantly. Ali and Puah [60] reported the insignificant negative impact of CR on Pakistani banking system. Their study using the multiple regression model based on the data of Pakistani banks found that higher NPL provisions arising from extreme lending decrease banks profitability. Haris, Yao [61] used GMM method on the sample of 33 Pakistan banks over the period of 2012 to 2016. Their study found a negative impact of CR on banking profitability. Karim, Akhtar [62] applied regression and GMM approaches and reported that CR has a significant and positive effects on the profitability of 40 banks. Their findings indicate the banking sector remained efficient during Covid-19.

Aluko, Kolapo [63] applied multiple regression-model and reported positive impact of CR and LR on bank profitability in the Nigerian banking sector from 2010 to 2016. Noman, Pervin [64] extracted data from 18 private banks of Bangladesh over the period 2003 to 2013 and applied panel regression, generalized least square (GLS) and GMM methods. They reported that CR has a negative and significant impact on ROA and ROE, but a positive impact on net interest margin (NIM). Sufian and Chong [65] applied the multiple regression analysis on Philippines banks over the period of 1990 to 2005 and their findings suggested that CR has a negative impact on bank profitability.

Gadzo, Kportorgbi [28] found that CR has a negative impact on profitability, which indicate that a higher ratio of NPLs causes a decrease in the profitability of 24 banks in Ghana. Tan, Floros [66] reported the negative impact of various risks factors including CR and LR on profitability of Chinese financial sector. Similarly Yahaya, Mahat [67] argued that NPLs have a negative impact on the profitability of financial institutions. They found that the NPLs are negatively related to the banking performance in term of profitability. Goswami and Malik [68] using a data of 75 Indian banks over the period 2018–2022 reported that lower LR increases the ROA and ROE while higher CR decreases the profitability of Indian banks.

#### 2.2.2 Findings on multiple economies

There are numerous studies available examining the impact of either CR, LR or both on the performance of banks operating in different economies. Such as, Saeed, Shahid [69] expressed that LR has a negative and significant impact on return-on-asset and return-on-equity. Their study applied GMM and the two-stage least square models to analyze the results by using a sample of Pakistani and Sri-Lankan banking industry during the year 2008 to 2016. Their results suggest that banks should keep higher reserves and use their assets efficiently to improving the performance of banking system. Le [70] investigated the association between bank liquidity and profitability. He reported that liquidity has a positive impact on banking profitability in twenty-eight European countries from 2010 to 2018. Ghenimi, Chaibi [71] reported a significant positive impact of LR on return on equity of banks in MENA region. They indicated that in order to gain additional profits, banks engage in taking high-risk, which rise the exposure of LR. However, in case of Islamic banks they reported that LR negatively effect the return on equity because the Islamic banks carry short-term loan portfolio of MENA region.

Abbas, Iqbal [72] used generalized method of moments (GMM) and multiple regression on data of US and Asian commercial banks from 2011 to 2017. They found that both LR and CR have a negative impact on profitability. Adusei [73] used multiple regression to analyze the data from 532 microfinance institutions in 73 countries and found that both LR and CR have a negative impact on performance. Yahaya, Mahat [67] used multiple regression to analyze the data of 50 Sub-Saharan institutions from 2011 to 2019 and found that both LR and NPLs have a negative and significant impact on ROA. They also found that higher loan commitments lead to higher NPLs, which reduces the profitability in the banking sector. Canh, Schinckus [74] used multiple regression approach and reported a negative impact of LR and CR on the bank profitability of banks in 56 economies over the period 2002 to 2015. Hunjra, Mehmood [75] reviewed the mixed results of LR and CR on ROA and ROE of 76 commercials banks of four Asian countries, i.e., Pakistan, Indian Bangladesh and Sri Lanka, over the period 2009 to 2018 using the GMM approach. Their results reported a negative impact of LR and CR on ROA and ROE. Abdelaziz, Rim [76] found a significant negative impact of CR and LR on profitability of 38 conventional banks in MENA countries over the period 2004 to 2015. Saleh and Abu Afifa [77] used the GMM approach to investigate the negative impact of LR and CR on banks profitability in emerging markets over the period 2010 to 2018.

Farihana and Rahman [78] applied the regression and GMM methods on a sample of 101 banks from 16 countries and found a negative and significant relation between profitability and CR in Islamic-banks over the period 2005 to 2012. Isayev and Bektas [79] used the one and two-step GMM techniques on 569 commercial banks in emerging economies over the period 2013 to 2018 and found an insignificant and positive effect of CR on banking profitability. Thornton and Di Tommaso [80] studied the positive and momentous impact between NPLs and profitability of 521 banks in 28 Europeans countries over the period of 2007–2017.

Concerning the evolution of performance in banking sector, Boussaada, Hakimi [81] reported the negative impact of NPLs on banks performance by using the sample of European banks from 2008 to 2017. The study suggested reduce NPLs are important and banks should pay more attention on policies to improving bank performance. Abbas, Iqbal [72] used GMM and two-stage least square approaches to compare the negative impact of CR on bank profitability in Asian commercial banks to that in US banks over the period 2011 to 2017. They found that the negative impact of CR on profitability is greater in Asian commercial banks. Hakimi, Boussaada [82] examined the negative impact of CR on banking profitability of conventional banks in MENA countries over the period 2004 to 2015. They found a significant negative relationship between CR and banking profitability. López-Penabad, Iglesias-Casal [83] reported the negative effect of interest-rate policy on profitability of 2596 banks in European countries over the period 2011 to 2019. They found that a decrease in NIM and assets ratio by 15.5 and 18.5 basis points, respectively, reduces bank profitability. Banks compensated for reduction in NIM by increasing fees and commissions.

#### 2.2.3 Risks and crises

It has been found that the banking risks trigger more during the crises period because of disturbance in the economic activities [62, 84]. Previously, Huang, Lan [85] reported that COVID-19 increased the financial risk in China. Yin, Han [84] reported that COVID-19 triggered the credit risk in Italy and Brazil. Li, Feng [86] reported that loan loss and deposits ratios decreased the profitability during COVID-19 of banks in US. Okpukpara, Okpukpara [10] that risk of loan default was higher during COVID-19 for the formal institutions in Nigeria. Ghenimi, Chaibi [7] reported that the COVID-19 negatively influenced the stability while positively influenced the financial risk of conventional banks in MENA region. Goswami and Malik [68] reported that during the first wave of COVID-19 the LR was higher for Indian banks. Karim, Shetu [87] reported that the liquidity position of banks in Bangladesh deteriorated, which triggered the liquidity risk. In addition, many studies were conducted on the liquidity and credit risk with regard to the sub-prime crisis, which reported that the sub-prime crisis triggered both types of risk [32, 57, 88–92]. Though the credit and liquidity risks play a vital role in the banking sector as other numerous previous studies have examined the relationship between LR and CR on banking profitability [25, 93–95]. However, no one has decisive evidence on the impact of LR and CR on profitability. As the Pakistan financial sector has always remain vulnerable to several crises, such economic, political, and financial which ultimately triggers different types of financial risk such as credit and liquidity risks, among others. Therefore, our study examines the impact of the most critical risks, i.e., credit and liquidity risk, on the profitability of Pakistani banking sector during and before COVID-19.

## 3. Data and methodology

### 3.1 Sample and data

The objective of this to explore the impact of LR and CR on the profitability of Pakistani banks during COVID-19. Therefore, our study is based on all Pakistani banks including commercial and microfinance banks. As per the central bank of Pakistan, 33 commercial banks, which are classified into public sector banks [5], private sector banks [20], specialized banks [4] and foreign banks [4], and 11 microfinance banks are operating in Pakistan (visit https://www.sbp.org.pk/publications/index2.asp). The data of this study is secondary in nature and obtained from the audited financial statements of banks on quarterly basis over the period Q12018 to Q42021. These statement are provided by each bank on their respective websites and the Central Bank of Pakistan. However, the financial reports of foreign banks including three other banks were not available. Thus, the final sample presented in Table 1 consists of 37 banks out of 45. In addition, out of 37 sample banks, some banks did not release their reports for some quarters. As a result, the data were unbalanced based on 576 bank-year observations. For the empirical analysis, the data is further divided into two periods, i.e., before COVID-19 period (Q2018 to Q42019) and during COVID-19 period (Q12020 to Q42021).

**Table 1:** List of samples.

Banks	Total FIs	Sample
Public Sector	5	4
Private Sector	20	19
Specialized	4	3
Microfinance	11	11
Total	40	37

### 3.2 Econometric methodology

This study examines the impact of the LR and CR on profitability using certain econometric techniques. The study used the STATA-17 for the econometric analysis. First, since the data of this study is based on unbalanced panels; therefore, the study applied fisher type Augmented Dickey Fuller (ADF) test to examine the unit root problem. The fisher type ADF unit root test is used to examine the stationarity among panels [96–98], especially when the data is unbalanced [4, 5, 99]. Second, a Variance Inflation Factor (VIF) test was applied to examine the multicollinearity problem. As the data of our study is based on unbalanced panels; therefore, we employed panel methodologies, i.e., pooled OLS and OLS Fixed and random models, for main analyses. The balanced panel data may suffer from the repeated cross-sectional time series issues, while the unbalanced panel data is free from such problems [100]. On the unbalanced data, the panel models such as OLS fixed and random effects are more useful because of giving less collinearity and high degree of freedom by allowing unobserved heterogeneity [100, 101]. Both fixed and random effects are better suited for unbalanced data as they allow us to account for the unobserved individual and time effects [101, 102] while the pooled OLS does not allow [103].

Though, the use of Pooled OLS and OLS (Fixed and random effects) with robust standard errors by handling the problems of serial correlation and heteroskedasticity produce robust and unbiased findings [5, 104]. However, if the used data suffers from the abnormality problem the obtained findings may become unreliable. Thus, the use of Quantile regression becomes significant if the data is not normally distributed [105]. The superiority of quantile regression is that it uses the same probability for offering the probability distribution ranges at different cut points, known as quantiles. The quantiles regression is also effective when the data is collected from different times [105, 106]. Further, it is also effective as it allows consider the impact of covariate on the full distribution of dependent variables, for offering the data’s completer characterization [105]. Therefore, for the robustness of our findings, we applied Quantile regression based on the Shapiro-Wilk normality test. Dealing with the data abnormality problem through the use of Quantile regression the study reported more robust and corrected findings. The regression equation of this study is as follows;

$${PROF}_{it}=\alpha \;+\;\beta_{1}{LR}_{it}+\beta_{2}{CR}_{it}+\;\beta_{3}{LNTA}_{it}+\;\beta_{4}{DIV}_{it}\;+\;\beta_{5}{FINS}_{it}+\;\beta_{6}{SOL}_{it}+\;\beta_{7}{OE}_{it}+\;\beta_{8}{AGE}_{it}+\;\beta_{s9}{OWN}_{it}+\;\varepsilon_{it}$$

In the above equation, PROF indicates the profitability, measured by ROA, ROE, and NIM. LR and CR represent the liquidity and credit risks, respectively. Further, LNTA, DIV, FINS, SOL, OE, AGE, and OWN represent the size, diversification, financial structure, solvency, operating efficiency, age of institutions and ownership, respectively. Apart from these, *α* is the constant term, *i* indicates individual-effect and *t* represents years-effect, *ε* is the error term and *β* is the coefficient.

### 3.3 Variable description

#### 3.3.1 Profitability

This study examines the impact of LR and CR on banking profitability of Pakistani banks. Three proxies are used to measure profitability, i.e., return on assets (ROA), return on equity (ROE), and net interest margin (NIM). ROA is calculated as post-tax profits divided by total assets. It measures how efficiently a bank generates revenues using its assets. ROE is calculated as post-tax profits divided by total equity. It measures how well a bank can use the funds invested by stakeholders to generate profit. NIM is the difference between the interest income earned from borrowers and the interest expense paid to depositors divided by total assets. It measures the profitability of a bank from its core lending activities.

#### 3.3.2 Liquidity risk

Liquidity risk (LR) in the banking sector is measured by the ratio of total loans to total deposits. A higher liquidity ratio indicates specifies a poor and unstable liquidity situation. Liquidity risk has also been studied in a number of previous studies [77, 107, 108]. The liquidity risk arises when banks are unable to fund the unanticipated demands arises from borrowers and depositors [30]. This liquidity risk has been found as a key determinants of bank profitability, influencing both positively and negatively [4, 32, 72, 75, 109]. During the COVID-19 pandemic, the liquidity risk in financial sector was triggered in an unprecedented manner. Ghenimi, Chaibi [7] indicated that COVID-19 increase the liquidity risk of banks in MENA region. Goswami and Malik [68] also reported that the LR was triggered during the first wave of COVID-19. However, the impact of LR on the profitability could be positive as previous studies reported the positive impact of LR on profitability because of its effective management. Banks may have the opportunity to earning more returns by charging higher interest in crisis because of unanticipated demand from borrowers. Previously, Haris, Yao [61], Molyneux and Thornton [110] and Sufian and Habibullah [109] have reported that the higher LR translates its positive impact on profitability of banks. Chen, Chen [32] argued that the impact of liquidity risk on the profitability during the crisis remains a debatable question. As LR could only be a symptom of banks insolvency [111]. Thus, its adverse impact can be controlled through having a control on CR. Moreover, the banks may adjust their assets and liabilities by having the less illiquid assets in order to cater the shocks from crisis [92]. Thus, the profitability will not suffer from LR in crisis.

#### 3.3.3 Credit risk

CR is defined as the loss that a bank tolerates when the borrowers fail to repay the loan amount upon agreed due date. CR is distinct from the risk of loss due to default or the inability of customers to meet short-term obligations on lending, trading, hedging settlements, and other financial agreements. The ratio of loan loss provisions to total gross loans is used to assess CR [112, 113]. The process of advancing or borrowing money is known as credit. A credit is generally classified as non-performing when amount of principal and interest are not recovered over 90 days or more. A higher CR indicates the poor credit management by a banks, which leads to lower profitability. To achieve higher profits, financial institutions use a higher-risk strategy [114]. Banks are required to keep more funds to deal with NPLs, which have documents negative impact on the profitability and causes decrease in the assets size of banks. A higher concentration of credit risk that occurs continuously can severely damage the financial system [115]. Several empirical studies have supported negative impact of CR on banks profitability, showing that a higher number of bad loans lead to lower profitability in banks [13, 28, 116]. The credit risk modeling theory access credit portfolio and help to manage bank risk [117].

The inverse relationship between CR and bank profitability is due to inefficient management of CR. Moreover, the higher investment by banks to increase their loan portfolio decrease the profitability because it may induce higher NPLs. Banks need to retain more capital to recoup bad debts, which decline collective magnitudes of assets and profits.

#### 3.3.4 Bank-specific variables

The study regressed some other bank-specific variables along with LR and CR. Bank-size is measured as the natural logarithm of total assets, is used to show the strength of bank. Large banks are more likely to generate more liquidity because they can create a diversified portfolio of assets and liabilities [118]. There are two opposing arguments regarding the relationship between bank size and liquidity. The first argument is “too-big to-fail”, which suggests that large banks are more likely to experience liquidity problems because they are more complex and interconnected. However, other argument proves that the banks broad in size gain more profit instead of smaller banks. Diversification is measured as total non-interest income divided by gross revenues. Non-interest income consists of fees, commissions, service charges, and other charges generated for banks. Higher level of diversifications can help reducing the ratio of loans to deposits, which improves the banks liquid position. Adzobu, Agbloyor [119] expressed that diversification has no effect on profitability and CR. Financial-structure is calculated as the ratio of bank deposits to bank equity. A growth in the level of deposits leads to higher ratio of funds, which are further converted into liquid assets according to the bank capability. Solvency is measured as total shareholder’s equity divided by total assets. Banks take the advantage of higher-ratio of equity to liabilities, which leads to an increase in profitability. Total operating expenses divided by gross income is used as a proxy to calculate the operational-efficiency. A high-level of operational efficiency expresses the ineffective utilization and inefficient management of operational expenses, which negatively impacts the profitability of banks profitability. Institutional ages is calculated as the natural logarithm of total years from the incorporation of a bank. The effect of ownership is controlled using a binary variable, which is equal to 1 if a bank is private-owned, and 0 otherwise. Table 2 presents the detailed list of variables.

**Table 2:** Variables description.

Variables	Notation	Description	Hypotheses
**Dependent**			
Return on assets	ROA	Profit after tax to total assets	+/-
Return on equity	ROE	Profit after tax to total equity	+/-
Net interest margin	NIM	Interest income—interest expense/total assets	+/-
**Independent**			
Liquidity risk	LR	The ratio of total bank loans to total deposits for a bank i in year t	+
Credit risk	CR	The ratio of loss-loan provisions to total gross loans	_
**Control**			
Bank size	SIZE	Natural logarithm of total assets	+
Bank Diversification	DIV	Total noninterest income/ gross revenue	_
Financial structure	FINS	Bank deposits/bank equity	+/-
Solvency	SOL	Total shareholder’s equity to total assets	+/-
Operational Efficiency	OE	Total operating expenses to gross income	+/-
Institution age	AGE	Natural logarithm of total years from incorporation	_
Ownership	OWNR	Dummy that equals to 1 if private-owned bank or 0	

## 4. Findings

### 4.1 Pre-estimation tests

The study applied two pre-estimation tests, i.e., unit-root and VIF, to ensure the accuracy of unbalanced panel data. The results of Augmented Dickey-Fuller (ADF) unit root test, which used to analyze the stationarity of data, are presented in Table 3. The ADF results indicate that our data does not suffer from unit root problem as the p-values of all coefficients are significant at 1% level and thus, making our data stationary [29]. The study applied Variance Inflation Factor (VIF) test to examine the level of multicollinearity and the results are mentioned in Table 4. The results depicted that the VIF values of all variables are less than the threshold of 10, indicating no multicollinearity among variables. Further, we also applied the correlation analysis to examine the correlation among used variables. The results reported in Table 5 show that the correlation coefficients of all variables are less than the threshold of 0.8 [120], also indicating no multicollinearity problem.

**Table 3:** Augmented Dickey-Fuller (ADF) unit root test.

Variables	Coeff	P-values
ROA	207.7258	0.0000
ROE	200.5540	0.0000
NIM	233.3064	0.0000
LR	290.1988	0.0000
CR	262.4640	0.0000
LNTA	297.1237	0.0000
DIV	211.0454	0.0000
FINS	164.6726	0.0000
SOL	182.6581	0.0000
OE	280.3147	0.0000
LNAGE	111.600	0.0031
OWNR	105.300	0.0000
TYPE	186.450	0.0000

**Table 4:** VIF test.

Variables	VIF
LR	1.22
CR	2.41
LNTA	4.94
DIV	1.08
FINS	1.30
SOL	2.87
OE	1.03
LNAGE	1.73
OWNERSH	1.38
TYPE	3.46
MEAN VIF	2.14

**Table 5:** Correlation matrix.

**Variables**	**ROA**	**ROE**	**NIM**	**LR**	**CR**	**LNTA**	**DIV**	**FINS**	**SOL**	**OPE**	**LNAGE**	**OWNR**	**TYPE**
ROA	1.000												
ROE	0.6917***	1.000											
NIM	0.134**	-0.0998**	1.000										
LR	0.134**	0.0071	0.2364***	1.000									
CR	-0.3971***	0.0985**	-0.3101***	-0.0390	1.000								
SIZE	0.2522***	0.2070***	-0.5313***	-0.2872***	-0.2246***	1.000							
DIV	-0.5288***	-0.5810***	0.0217	-0.0009	-0.0561	-0.1335**	1.000						
FINS	0.1737**	0.3027***	-0.112**	-0.1198**	-0.2591***	0.3826***	-0.0827	1.000					
SOL	0.2294***	-0.1551**	0.5021***	0.3720***	-0.4866***	-0.4048***	0.0640	-0.1661**	1.000				
OE	-0.1211**	0.1241**	-0.0825	-0.0072	0.0412	-0.0117	0.0397	-0.1106**	-0.0590	1.000			
LNAGE	0.0949	0.1141**	-0.2881***	-0.1352**	-0.0672	0.5473***	0.1039**	0.0967**	-0.0379	-0.046	1.000		
OWNR	0.0015	-0.0287	-0.1872**	-0.0536	0.0335	0.2086***	-0.0972	0.0382	-0.0131	-0.021	0.0894	1.000	
TPYE	-0.2084***	-0.2712***	0.6338***	0.1985**	-0.0133	-0.7603***	0.2408***	-0.2746***	0.356***	-0.038	-0.4529***	-0.4483***	1.000

**Notes:** In this table *’ **’ ***’ indicates the level of significance 10%, 5% and 1%. This table examine the correlation coefficient between the independent variable and check whether the multicollinearity problem exist in the data or not.

#### 4.1.1 Descriptive statistics

Descriptive statistics depicted the main attributes of data. Table 6 present the mean and standard deviation values of all the banking sector and a comparison between PPB, PCB, PSB and PMB during the period of 2019 to 2022. The mean value of LR and CR in the overall Pakistani banking system found 5.57% and 0.19%, respectively. However, the mean value of LR is 43.33% in case of PPB, 53.3% case of PCB, 147.7% in case of PSB, and 176.66% in case of PMB. Further, mean value of CR is 0.2287 in case of PPB, 0.100 in case of PCB, 1.269 in case of PSB and 0.032 in case of PMB. The results reported that the mean value of ROA in Pakistani banking sector is -0.002, ROE is 0.036 and 0.031 is NIM during the analysis-period. Similarly, the mean values of ROA for PPB and PCB are 0.0024 and 0.003 respectively, while the mean values of ROA for PSB and PMB are -0.028 and -0.008 respectively. It indicates that the banking sector of Pakistan facing poor condition of ROA. The negative ROA indicates the lower profit earned in comparison to their total assets [121]. The mean values of ROE for PPB, PCB, and PSB are positive, while mean ROE for PMB is negative (-0.0089), which indicates that the microfinance banks in Pakistan are facing lower profitability when it comes to ROE. The mean value of NIM is 0.013 in case of PPB, 0.018 in case of PCB, 0.014 in case of PSB and 0.065 in case of PMB. It indicates that the Pakistani banks experienced profitability in terms on NIM during the period under analysis.

**Table 6:** Summary statistics.

	TPB			PPB		PCB		PSB		PMB	
Variables	Obs	Mean	S. D	Mean	S. D	Mean	S. D	Mean	S. D	Mean	S. D
ROA	293	-0.0024	0..0290	0.0024	0.0053	0.003	0.0094	-0.028	0.0360	-0.0085	0.0457
ROE	293	0.0367	0.2677	0.0433	0.0848	0.106	0.1529	0.0357	0.1367	-0.0896	0.4177
NIM	293	0.0310	0..0316	0.0136	0.0081	0.018	0.011	0.0143	0.0250	0.0654	0.0370
LR	293	5.5693	35.749	0.4333	0.1363	0.533	0.1637	1.477	0.9299	17.663	65.067
CR	293	0.1903	0.5745	0.2287	0.4286	0.100	0.2181	1.269	1.5035	0.0318	0.0478
LNTA	293	19.154	1.9463	20.420	1.042	20.33	1.005	17.530	1.312	17.018	1.3997
DIV	293	0.1774	0.3198	0.0952	0.053	0.134	0.06011	0.0847	0.1069	0.3105	0.5645
FINS	293	8.8723	10.402	12.116	3.505	11.39	13.144	-0.389	1.444	5.7642	3.275
SOL	293	0.1054	0.1510	0.0637	0.0163	0.060	0.0429	0.1716	0.3905	0.1830	0.1500
OE	293	1.8540	20.123	0.6816	0.3288	3.280	27.811	-1.942	5.2888	0.8164	0.3587
LNAGE	293	3.1348	0.6508	3.371	0.6929	3.332	0.5694	3.356	0.5971	2.6294	0.5004
OWNR	293	0.1365	0.3439	1	0	0	0	0.3333	0.4815	0	0
TYPE	293	2.552	1.0244	1	0	2	0	3	0	4	0

**Notes:** TPB indicates the total Pakistani banks; PPB Pakistani public banks; PCB Pakistani commercial banks; PSB Pakistani specialized banks; PMB Pakistani microfinance banks. In this table the data of 37 Pakistani banks are available. We have taken the data of two years (2020–2022). Quarterly base data is used in this study. The total numbers of observation are 293 but some banks statements are not published over the period of time so the 3 observations are not available in data.

### 4.2 Regression results

Tables 7–10 present the empirical results of the study. We divided the data into two different panels based on before COVID-19 period and during COVID-19 period to offer the comparative findings. Table 7 shows the results of the pooled OLS regression model. The F-statistics are highly-significant (p < 0.01), indicating that all models are well-fit for the data. The R-squared values ranging from 0.199 to 0.5896 show that the independent variables explain up to 59% of the variations in the dependent variables. The coefficients of LR are positive and significant (β = 0.0005, p<0.01; β = 0.0005, p<0.01), indicating that a 1% increase in LR leads to a 0.0005 increase in ROA during COVID-19 and lead to 0.0003 increase in ROA before COVID-19. Though, the coefficients of LR for ROE are positive both during and before COVID-19 but this impact is only significant during COVID-19 (β = 0.0003, p<0.01), indicating that during COVID-19 a 1% increase in LR leads to a 0.0003 increase in ROE. The coefficients of LR for NIM are negative but not significant (β = -0.0003, p>0.10; β = -0.00001, p>0.10), indicating that a 1% increase in LR does not have any significant impact on NIM both during and before COVID-19 periods. Though, the impact of CR is positive but this impact is low because the beta coefficients of LR are small in each model. The coefficients of CR for ROA are negative and significant (β = -0.119, p<0.01; β = -0.0330, p>0.01), indicating that a 1% increase in CR leads to a 0.119 and 0.0330 decrease in ROA both during and before COVID-19 periods, respectively. Though, the coefficients of CR for ROE are negative (β = -0.0968, p<0.01; β = -0.0417, p>0.10) but it indicates that a 1% increase in CR leads to a 0.0968 decrease in ROE during COVID-19 period only because CR shows an insignificant impact on ROE before COVID-19. The coefficients of CR for NIM are negative and significant (β = -0.018, p<0.01; β = -0.0259, p<0.01), indicating that a 1% increase in CR leads to a 0.018 and 0.0259 decrease in NIM both during and before COVID-19 periods, respectively.

**Table 7:** Results of pooled OLS.

	During COVID-19			Before COVID-19		
Variables	ROA	ROE	NIM	ROA	ROE	NIM
LR	0.0005***	0.0003***	-0.0003	0.0003***	0.00004	-0.00001
CR	-0.0619***	-0.0968***	-0.018***	-0.0330***	-0.0417	-0.0259***
SIZE	0.0045***	0.0123	-0.00425***	-0.0020	0.0268*	-0.0060***
DIV	-0.0473***	-0.4501***	-0.0137***	-0.0000	0.0009	-0.0003**
FINS	-0.0000	0.0076***	0.0001	0.0002	-0.0306***	0.0003
SOL	0.0491***	0.1672	0.0113	-0.0103	-0.1844**	0.0254***
OE	-0.0001**	0.0022***	-0.00005	0.0003	-0.0072**	0.00001
AGE	-0.008***	-0.01511	0.0017	0.0047*	-0.0216	0.0038
OWNR	-0.011**	-0.1231***	0.0079**	-0.0109*	-0.1003**	0.0166***
TYPE	-0.003	-0.0298	0.0154***	-0.0009	-0.0706***	0.0186***
F	32.43***	25.93***	36.66***	8.00***	18.77***	41.51***
AJD R2	0.5184	0.4605	0.5498	0.1990	0.3866	0.5896
N	293	293	293	283	283	283
CONSTANT	-0.0514**	-0.0905	0.0703**	0.3045	0.0994	0.0853***

**Notes:** This tables shows the results of pooled OLS it examines the relationship between the variables. In this table * ‘**’*** suggested the 10%, 5% and 1%, respectively.

**Table 8:** OLS random and fixed effect.

	ROA				ROE				NIM			
	BEFORE COVID-19		During COVID-19		BEFORE COVID-19		During COVID-19		BEFORE COVID-19		During COVID-19	
	RE		FE		FE		FE		RE		FE	
Variables	Coeff	t-value	Coeff	z-value	Coeff	t-value	Coeff	z-value	Coeff	t-value	Coeff	z-value
LR	0.00001	1.05	0.00003***	2.75	0.00003	0.18	0.00004**	2.08	-0.0001**	-2.16	0.0001	1.01
CR	-0.0409***	-5.33	-0.0026***	-3.34	-0.0888	-0.52	-0.0020***	-3.03	-0.0241***	-3.70	-0.0127***	-3.54
SIZE	-0.0006	-0.27	0.0264***	4.11	0.0131	0.41	0.0674	1.16	-0.0038*	-1.86	0.0527***	7.57
DIV	-0.0000	-0.38	0.0047	0.87	0.0001	0.10	0.0707	1.44	-0.0002**	-1.97	-0.0102**	-1.74
FINS	0.0001	0.78	0.00002	0.19	-0.0436***	-20.04	0.0107***	9.79	0.0001	0.51	-0.00005	-0.43
SOL	-0.0083	-1.28	0.0566	1.32	0.0242	0.30	0.3086	0.79	0.0346***	4.87	-0.2901***	-6.20
OE	0.0002	0.79	-0.0001***	-2.81	-0.0053**	-1.95	0.0011**	2.47	0.0000	0.37	-0.00004	-0.74
AGE	0.0009	0.16	-0.0489***	-1.87	0.1049	0.29	-0.2889	-1.22	0.0004	0.10	-0.148***	-5.20
OWNR	-0.0101	-0.99	Yes		Yes		Yes		0.0174**	2.22	Yes	
TYPE	-0.0009	-0.19	Yes		Yes		Yes		0.0201***	5.06	Yes	
CONSTANT	0.0165	0.33	-0.3612***	-3.56	-0.1350	-0.11	-0.492	-0.54	0.0502	1.11	-0.4852***	-4.39
F/WALD	35.88***		5.09***		53.53***		13.71***		173.16***		9.55***	
NN	283		293		283		293		283		293	
ADJ-R2	0.1058		0.1411		0.6428		0.1411		0.1568		0.2355	

**Notes:** This table shows the result of random and fixed effect model from 2020 to 2022. These results indicate the relationship between the profitability and risk. The results indicate *’ **’ *** the level of significance.

**Table 9:** Shapiro-Wilk W test for normal data.

		Before COVID-19					During COVID-19			
Variables	Obs.	W	V	Z	Prob>z	Obs.	W	V	Z	Prob>z
ROA	283	0.5501	91.024	10.559	0.0000	293	0.5067	102.93	10.86	0.0000
ROE	283	0.3983	121.75	11.240	0.0000	293	0.4683	110.93	11.04	0.0000
NIM	283	0.7652	47.503	9.037	0.0000	293	0.8249	36.53	8.436	0.0000
LR	283	0.0638	189.414	12.275	0.0000	293	0.1254	182.47	12.21	0.0000
CR	283	0.2751	146.664	11.676	0.0000	293	0.3110	143.75	11.65	0.0000
SIZE	283	0.9377	12.604	5.931	0.0000	293	0.9465	11.16	5.657	0.0000
DIV	283	0.0385	194.538	12.337	0.0000	293	0.3634	132.83	11.46	0.0000
FINS	283	0.7869	43.107	8.810	0.0000	293	0.4923	105.93	10.93	0.0000
Sol	283	0.4152	118.323	11.173	0.0000	293	0.7010	62.39	9.691	0.0000
OE	283	0.1506	171.862	12.047	0.0000	293	0.0662	194.81	12.36	0.0000
AGE	283	0.9596	8.172	4.917	0.0000	293	0.9595	8.455	5.005	0.0000
OWNR	283	0.9702	6.026	4.204	0.0000	293	0.9618	7.963	4.865	0.0000
TYPE	283	0.9639	7.293	4.651	0.0000	293	0.9668	6.938	4.542	0.0000

**Notes:** Shapiro-Wilk test applied on data to check the normality of data. The observation is used in this study in the number 293 during COVID-19 and 283 before COVID-19.

**Table 10:** Quantile regression (ROA).

	Before COVID-19					During COVID-19				
Variables	0.5	0.25	0.50	0.75	0.90	0.5	0.25	0.50	0.75	0.90
LR	0.0001	0.0001**	0.00002**	1.54	-0.00001*	0.0001	0.0001**	0.00003**	0.0001***	0.0001***
CR	-0.1169***	-0.0413***	-0.0239***	-0.0165***	-0.0090***	-0.0153***	-0.0217***	-0.0145***	-0.0027	-0.0023
LNTA	0.0021	-0.0007	0.0007	0.0019***	0.0018***	0.005***	0.0033**	0.0030***	0.0037***	0.0032***
DIV	-0.0003	-0.0000	-0.0000	-0.0001	-0.0001**	-0.096***	-0.073***	-0.018***	-0.006***	-0.007***
FINS	2.74	0.0002	2.22	-0.0001	-0.0003***	-0.0001	0.0000	0.0000	0.00001	4.70
SOL	0.0037	-0.0150**	0.0005	0.0136***	0.0105***	0.0414**	0.014	0.0185**	0.0505***	0.0548***
OPE	0.0001	0.0002	-0.0012***	-0.0019***	-0.0022***	-.0000	-0.0001	-0.0001***	-0.0001***	-0.0001***
LNAGE	0.0046	0.0051**	0.0016	-0.0013	-0.0028***	-0.0056**	-0.0008	-0.0024**	-0.004***	-0.0023**
OWNR	-0.0361**	-0.0123***	0.0010	0.0054**	0.0066***	-0.019***	-0.005	-0.001	0.00004	-0.0001
TYPE	-0.0213**	0.0020	0.0030***	0.0063***	0.0080***	-0.006**	-0.0003	0.0014	0.0020**	0.0030**
CONSTANT	-0.0124	-0.0032	-0.093	-0.0375***	-0.0292**	-0.075**	-0.0538	-0.015***	-0.060***	-0.056***
ADJ-R2	0.6225	0.2301	0.1377	0.1661	0.2769	0.6225	0.4159	0.2408	0.1911	0.2706

**Notes:** The results indicate *’ **’ *** the level of significance.

Though the pooled OLS offers reliable findings but due to individual and time-varying effects the results based on pooled OLS could be unreliable. Therefore, we have applied OLS fixed and random effect models to offer more reliable findings. The appropriate selection between fixed and random effect models was performed using the Hausman [122] test for each model presented in Table 8. The values of F/Wald-statistics are highly significant (p<0.005), which reveal that our models are good-fit. The value of R-squared affirms that the independent variables explain the variability in the profitability of banks. The results show that the coefficients of LR are positive but insignificant for ROA (β = 0.00001, P>0.10) and ROE (β = 0.00003, P>0.10) before COVID-19 while, the coefficients of LR are positive and significant for ROA β = 0.0003, p<0.01) and ROE (β = 0.00004, p<0.05) during COVID-19. The results of LR are consistent with Gadzo, Kportorgbi [28]. Further, results report that before COVID-19, the coefficients of CR are negative and significant only for ROA (β = -0.0409, p<0.01) and NIM (β = -0.0241, p<0.01), while during the COVID-19, the NIM reports its negative and significant coefficients for ROA (β = -0.0026, p<0.01), ROE (β = -0.002, p<0.01) and NIM (β = -0.0127, p<0.01). The negative impact of CR on profitability is consistent with Thornton and Di Tommaso [80]. The higher CR suggests that the banks are offering risky loans to their borrowers. Those low quality loans increase the ratio of NPLs, which decrease the profitability of banks. The higher the CR cause low profitability Haris, Yao [123].

#### 4.2.1 Additional robust check

**Fig 1:**
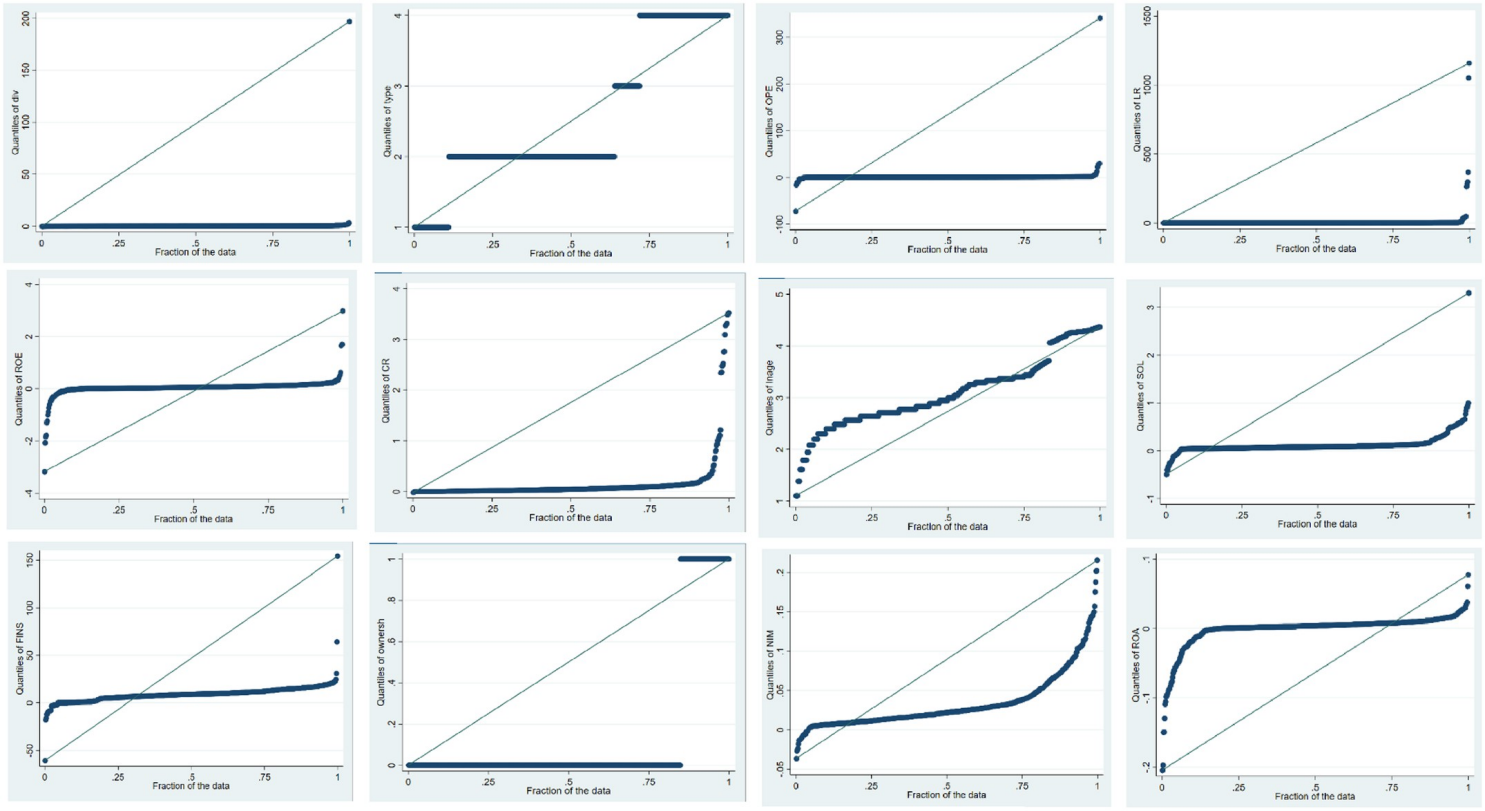
Histogram of data normality of all variables.

Though the results of this study are robust to Pooled OLS and OLS fixed and random effect methods but we applied Quantile regression as a robust check. It handled the data abnormality and offered more reliable and corrected statistics. Shapiro-Wilk test was applied to check the data normality, which is reported in Table 9 and based on the p-values<0.05 it was found that the data is not normally distributed. This is also evident in the graphical representation presented in Fig 1. The Fig 1 shows the histogram of data normality of all variables, which shows that the data is not bell-shaped and hence, suffers from normality issues.

Table 10 presents the results of ROA based on quantile regression. During the COVID-19, the coefficients of LR are significant and positive at 0.25^th^, 50^th^ and 90^th^ quantiles, while during COVID-19, its all coefficients are positive and significant at all quartiles except 0.5^th^. The results with regard to LR and ROA are consistent with the results of pooled OLS and fixed effects. The results between CR and ROA show that, before the COVID-19, the coefficients of CR are negative and significant at all quantile levels, while during the COVID-19, the coefficients are negative and significant at three quantiles only, i.e., 0.5^th,^ 0.25^th^, 0.50^th^.

The results reported in Table 11 show that before COVID-19, the coefficients of LR are positive but insignificant at all quantile levels while during the COVID-19, the coefficients of LR are positive and significant at 25^th^ and 50^th^ quartiles. The results also depict that the before COVID-19, the negative and significant relationship between CR and ROE exist at all quantile levels except 0.25^th^ while during COVID-19, the negative relationship between CR and ROE exist only at 50^th^, 75^th^ and 90^th^ quantiles.

**Table 11:** Quantile regression (ROE).

	Before COVID-19					During COVID-19				
Variables	0.10	0.25	0.50	0.75	0.90	0.5	0.25	0.50	0.75	0.90
LR	0.0002	0.00003	-0.0001	-0.00001	-0.0001	0.0008	0.0007**	0.0004**	0.0003	0.0003
CR	-1.0332**	0.0407	-0.0777***	-0.1453***	-0.2155**	0.0380	0.0371	-0.0698***	-0.0825***	-0.1222***
LNTA	0.0333	0.0088	0.0178***	0.0385***	0.0446	0.0244	0.0308**	0.0244***	0.0184**	0.0072
DIV	0.0017	-0.000	-0.0002	-0.0003	-0.0001	-0.922***	-0.896***	-0.122***	-0.0504**	-0.0057
FINS	-0.0211	-0.0008	-0.0017**	-0.0050***	-0.0183***	0.001	0.0007	0.0009	0.005***	0.0102***
SOL	0.0513	-0.0601	-0.0210	-0.0477	-0.0942	0.0843	0.0280	0.0269	0.0106***	-0.0262
OPE	-0.0269	-0.0222***	-0.0213***	-0.0191***	-0.0122**	0.0024**	0.0022***	0.0016***	0.0105***	0.0022***
LNAGE	0.0570	0.0136	-0.0130	-0.0401**	-0.0287	0.0082	-0.0019	-0.0207	-0.0269**	-0.0287
OWNR	-0.0906	-0.0479***	0.0046	0.0437	0.0164	-0.163**	-0.117***	-0.0520**	-0.0445**	-0.0642
TYPE	-0.0511	0.0134	0.0208**	0.0434***	0.0001	-0.0278	0.003	0.0058	0.0037	-0.0081
CONSTANT	-0.5656	-0.1912	-0.2694***	-0.5679***	-0.3335	-0.3713	-0.4858**	-0.363**	-0.2208	0.0306
ADJ-R2	0.2414	0.0981	0.1053	0.1378	0.2196	0.5002	0.2375	0.1490	0.1839	0.3142

**Notes:** The results indicate *’ **’ *** the level of significance.

In Table 12, the results of LR reported positive coefficients at 0.5^th^ quantile and negative at 0.90^th^ quantile while during the COVID-19, the coefficients of LR are positive at 0.5^th^, 0.25^th^ and 0.50^th^ levels. The coefficients of CR before the COVID-19 are also negatively associated with NIM at all quartiles with values of -0.0161, -0,0129, -0.019, -0.0284 and -0.0215 at the 5^th^, 25^th^, 50^th^, 75^th^ and 90^th^ quantiles, respectively. However, the coefficients of CR for NIM during COVID-19, are only positive and significant at 0.5^th^, 0.25^th^, 0.50^th^ and 75^th^ quantiles with -0.0116, -0.0163, -0.0156 and -0.0075, respectively. All the coefficients of LR and CR for ROA, ROE and NIM are statistically significant at p<0.01 and p<0.05 levels at all quantiles.

**Table 12:** Quantile regression (NIM).

	Before COVID-19					During COVID-19				
Variables	0.5	0.25	0.50	0.75	0.90	0.5	0.25	0.50	0.75	0.90
LR	0.00004***	6.48	-0.00002	-0.00002	-0.0001***	0.0001***	0.0001***	0.0002***	0.0001	-0.0001
CR	-0.0161***	-0.0129***	-0.0190***	-0.0284***	-0.0215***	-0.0116***	-0.0163***	-0.0156***	-0.0075**	-0.0044
LNTA	0.0010**	-0.0014	-0.0007	0.0000	-0.0003	0.0003	-0.0003	0.0014	-0.0003	-0.0044
DIV	0.0000	-0.0001	-0.0002*	-0.000***	-0.0004**	-0.0069	-0.0114***	-0.0177***	-0.023***	-0.029***
FINS	0.0000	0.0003	0.0001	-0.0003	0.000	0.0001***	0.0001	0.0000	4.87	0.000
SOL	0.0245***	0.0310***	0.0266***	0.0248***	0.085***	0.0024	-0.0173	-0.0054	0.0758***	0.146***
OPE	0.0000	0.0001	0.0002	0.0002	0.0001	-0.00001	-0.0000	-0.00004	-0.0000	-0.00004
LNAGE	-0.000	0.0018	0.0006	- 0.0014	-0.0009	0.000	0.0007	-0.0013	0.0005	0.006
OWNR	0.0008	0.0081*	0.0220***	0.0359***	0.038***	0.0065***	0.0098**	0.0132**	0..0179**	0.017**
TYPE	0.0006	0.0095***	0.0248***	0.0365***	0.038***	0.0089***	0.0140***	0.0201***	0.026***	0.0234***
CONSTANT	-0.0163	0.0078	-0.0225	-0.0412	0.0409	-0.0188	-0.0111	-0.0424	0.4072***	0.050
ADJ-R2	0.2760	0.1944	0.3322	0.5322	0.6332	0.3573	0.2813	0.3087	0.4	0.5627

**Notes:** The results indicate *’ **’ *** the level of significance.

## 5. Discussion

This study examines the impact of LR and CR on profitability over the period of Q12018-Q42021 by dividing the time period into two different panels, i.e., before and during COVID-19. The findings based on the pooled OLS, OLS fixed and random effects models and quantile-regression shed light on the impact of LR and CR on banking profitability.

The results show that the LR has reported its significant positive impact on profitability, which indicates that the higher liquidity risk increased the banking profitability during and before COVID-19. However, the impact of LR on profitability is higher during COVID-19 than before COVID-19. This could be because of the fact that during the COVID-19, on the one hand, due to the slower economic activities the borrowers were unable to repay their loans, which triggered the liquidity concerns for banks. While on the other hand, due to the loan restructuring for existing borrowers and demand for high loans from the new borrowers because of the high working capital shortages the banks were in an opportunist position to earning good margins. Consequently, the profitability responded positive to the increased liquidity risk. The positive relationship between LR and profitability is supported by the risk theory of profit of Hawley [124] and the traditional risk-return theory that banks earns more margins because of assuming more risk. Moreover, positive relationship is consistent with Le [70], Ghenimi, Chaibi [71], and Karim, Akhtar [62], while contrary to Saeed, Shahid [69], Canh, Schinckus [74], Hunjra, Mehmood [75] and Adusei [125]. The banks are involved in higher risk-taking activities to fascinate the clients towards investment which causes a rise in prolonged liabilities. While comparing our results to studies where the management of COVID-19 was not effective, such as Li, Feng [86] used the during COVID-19’s data of US banks and reported that the liquidity measured as the deposit ratio had a negative impact on ROE. El-Chaarani, Ismail [126] reported that the liquidity risk increased the performance of banks in GCC countries but the magnitude of the positive impact of LR and profitability fell down during COVID-19 period.

Higher LR raised the banks CR, which depicted higher ratio of NPLs because the borrowers could not repay at due time during COVID-19. Consequently, banks faced failure to making profits. The empirical findings indicate that during crisis the banking sector faced a reduction in credit demand and transitional business as well as an expansion of deferred advances (NPLs), which impacted the liquidity of banks [25, 32, 81, 85]. Concerning to our findings, the CR has reported its significant and negative impact on ROA, ROE and NIM during and before COVID-19. Though, the effect of CR on profit is negative but comparing with before COVID-19 period the negative effect is little lower during the COVID-19 period. This could be because of the fact that unlike the other countries Pakistan has imposed smart lockdown on economic activities. Pakistan is among only few countries who managed COVID-19 well and faced less risk. Along with smart lockdown strategy, central banks of Pakistan directed the banks to restructure the existing loan contracts to make borrowers able to repay their debts over the extended period. While, at the same time it has given an opportunity to earning more interest and thus raising the concerns for credit risk but with better profits. A classical study argued that the issuance of more risky loans that later convert into NPLs has a negative impact on banking profitability [127]. However, few studies argued that the effect of credit risk on banks stability could be minimize through its effective management [52, 53]. Similarly, Goswami and Malik [68] argued that the effective measures taken by the Central Bank and Indian government in combating the pandemic situation reduce the risk of defaults for banks. However, the negative relationship between CR and profitability is consistent with numerous studies such as Boussaada, Hakimi [128], Abdelaziz, Rim [76], Gadzo, Kportorgbi [28], Bester [129], Ekinci [130], Saleh and Abu Afifa [77] and Tan, Floros [66], while contrary to Aluko, Kolapo [63] and risk-return hypothesis. While, comparing our study with another countries where the COVID-19 was not managed effectively, such as Li, Feng [86] reported a very high magnitude of the negative impact of CR on ROA and ROE in US banks. El-Chaarani, Ismail [126] reported the negative impact of CR and profitability during COVID-19 period with a high magnitude of banks in GCC countries. Additionally, Huang, Lan [85] reported that the COVID-19 increased the systematic financial risk of Chinese banking sector and Yin, Han [84] reported that COVID-19 increased the credit risk in Brazil and Italy. Moreover, the contradictory results of other studies with regard to LR and CR are mainly because of the regional, periodical and methodological differences and also due to the use of different proxies of LR along with different combinations of independent and control variables.

## 6. Conclusion

This study is the first to analyze the impact of LR and CR on bank profitability, represented by three measures, i.e., ROA, ROE, and NIM, during and before the COVID-19. The sample consists of 37 Pakistani banks and the sampled data were analyzed by applying Pooled OLS, OLS fixed and random effects models, and quantile regression techniques. This study finds that LR has a positive impact on ROA, ROE and NIM during and before the COVID-19. This is likely due to the fact that banks with higher LR tend to earning more profits due to the extensive demand for loans from the borrowers because of working capital shortages that triggered from economic downturn during crises. The higher NPL ratio observed in our empirical results may also be contributing to the low margins, as NPLs represent loans that are unlikely to be repaid. Therefore, study also finds that CR has a negative impact on ROA, ROE and NIM during and before COVID-19. This is likely due to the fact that banks with higher CR ratios are more resilient to shocks, but they also have to hold more capital, which reduces their ability to generate profits. However, during the COVID-19, we found the low impact of CR on profitability and this could be because of the extension in credit terms through loan restructuring.

Our study’s findings are useful for academic, banks supervisors and regulators to framing the policies for risk management. It is evident from the literature that crises trigger credit risk with more speed but our findings suggest that, if managed-well, the effect of crises could be minimized. Alike the recent financial crisis 2007–2008, during COVID-19 the world has witnessed the significant role of central banks and regulators around the globe to keep financial system from failing such as the central bank of Pakistan directed the banks to restructure the loan in order to minimize the effect of COVID crisis on loan default and thus managed the credit risk well. It is therefore, suggested through our study that the role of regulators is very significant during the crises for the effective risk management through introducing loan restructuring schemes such as extending loan tenure, loan refinancing, easy collateral conditions, and subsidized funding cost. More specifically the regular and prompt follow-up for loan recovery is important to minimizing the risk of default and increasing the bank liquid position to combat crises. Further, with regard to LR, it is also suggested to keep greater amount of equities plus liabilities into short-term assets in order to fulfill the sudden need of liquidity under crises. Additionally, banks should take proactive measures beyond regulatory requirements, such as minimum liquidity under Basel III accord, by keeping long-term liabilities in terms of fixed or less liquid deposits. Our study also supported the risk-return theory that on the one hand though the risk such as liquidity risk raises the concern for returns. While, on the other hand if managed-well, it creates the opportunity to earning more returns. Our study finally endorsed the Basel III frameworks that both credit and liquidity risks are the most important factors that influence profitability and thus, their joint management is utmost important for keeping banking system away from collapsing.

Though the results of our study are robust to several econometric methodologies, it has certain limitations. Our study is based on the Pakistani banking sector and therefore, the future studies may examine that how the liquidity and credit risk affected the profitability of banks operating in other emerging economies. Due to the difference in political structure of countries, the management of COVID-19 was also different; therefore, the future studies may examine the role of political structure while examining the relationship between risk and profitability. Further, during the COVID-19, the monetary policy responded very positively to ensuring the stability of banking sector. Thus, it is recommended that future studies may examine the role of monetary policies issued during COVID-19 to see how their tools were useful for ensuring the stable profitability of the banking sector.

## Supporting information

S1 Data (XLSX)

S1 Appendix (DOCX)
