# Correction: The impact of liquidity risk and credit risk on bank profitability during COVID-19

**DOI:** 10.1371/journal.pone.0334269

**Published:** 2025-10-10

**Authors:** Muhammad Haris, HongXing Yao, Hijab Fatima, and the PLOS One Editors

After publication of this article [1], the following errors were identified in the References list:

- The article cited as Reference 2 in the published article is incorrectly duplicated as References 61, 99, and 123.
- The article cited as Reference 19 in the published article is incorrectly duplicated as References 30 and 63.
- The article cited as Reference 31 in the published article is incorrectly duplicated as Reference 116.
- The article cited as Reference 44 in the published article is incorrectly included in the References list and should not have been cited.

Additionally, References 21, 25, 27, 73, and 80 are incomplete. Please see the corrected references below:

- Reference 21, cited below as [2]: Naili M, Lahrichi Y (2022) Banks’ credit risk, systematic determinants and specific factors: recent evidence from emerging markets. Heliyon 8(2): e08960. https://doi.org/10.1016/j.heliyon.2022.e08960
- Reference 25, cited below as [3]: Kesraoui A, Lachaab M, Omri A (2022) The impact of credit risk and liquidity risk on bank margins during economic fluctuations: evidence from MENA countries with a dual banking system. Applied Economics 54(35): 4113–4130. https://doi.org/10.1080/00036846.2021.2022090
- Reference 27, cited below as [4]: Zolkifli N, Hamid M, Janor H (2015) Liquidity Risk and Performance: The Case of Bahrain and Malaysian Banks. Global Economy and Finance Journal 8(2): 95–111. https://doi.org/10.21102/gefj.2015.09.82.07
- Reference 73, cited below as [5]: Adusei M (2022) The liquidity risk–financial performance nexus: Evidence from hybrid financial institutions. Managerial and Decision Economics 43(1): 31–47. https://doi.org/10.1002/mde.3357
- Reference 80, cited below as [6]: Thornton J, Di Tommaso C (2021) The effect of non-performing loans on credit expansion: Do capital and profitability matter? Evidence from European banks. International Journal of Finance & Economics 26(3): 4822–4839. https://doi.org/10.1002/ijfe.2042

The *PLOS One* Editors note that they were unable to locate or verify Reference 27 [4] using the additional information provided by the authors.

The authors apologize for the errors in the article.
