# Supplementary material for: The impact of liquidity risk and credit risk on bank profitability during COVID-19
Source: PLoS One. 2024 Sep 9;19(9):e0308356. doi: 10.1371/journal.pone.0308356 (PMC11383245; doi:10.1371/journal.pone.0308356)
Supplement: S1 Appendix — (DOCX) [file pone.0308356.s002.docx]

**Appendix A. List of Abbreviations**

Profitability PROF

Return on Assets ROE

Return on Equity ROE

Net Interest Margin NIM

Liquidity Risk LR

Credit Risk CR

Natural Logarithm of Total Assets LNTA

Bank Diversifications DIV

Financial Structure FINS

Solvency SOL

Operational Efficiency OE

Institution Age AGE

Ownership OWN

Non-performing Loans NPLs

Ordinary Least Square OLS

Generalized Least Square GLS

Generalized Methods of Moments GMM

Augmented Dickey Fuller ADF

Variance Inflationary Factor VIF

Total Pakistani Banks TPB

Pakistani Public Banks PPB

Pakistani Commercial Banks PCB

Pakistani Specialized Banks PSB

Pakistani Microfinance Banks PMB
